# Immunomodulatory effects of metronomic vinorelbine (mVRL), with or without metronomic capecitabine (mCAPE), in hormone receptor positive (HR+)/HER2-negative metastatic breast cancer (MBC) patients: final results of the exploratory phase 2 Victor-5 study

**DOI:** 10.1186/s12885-022-10031-6

**Published:** 2022-09-06

**Authors:** F. F. Pepe, M. E. Cazzaniga, S. Baroni, F. Riva, F. Cicchiello, S. Capici, V. Cogliati, C. Maggioni, N. Cordani, M. G. Cerrito, S. Malandrin

**Affiliations:** 1 Phase 1 Research Centre, ASST Monza, Monza, Italy; 2 School of Medicine and Surgery, University of Milano Bicocca, Monza, Italy; 3 Oncology Unit, ASST Monza, Monza, Italy; 4 Microbiology Unit, ASST Monza, Monza, Italy

**Keywords:** Metronomic chemotherapy, Treg, Vinorelbine, Breast Cancer

## Abstract

Tregs are able of suppressing tumor-specific effector cells, such as lymphocytes CD8+, CD4+ and Natural Killer cells. Different drugs, especially different schedules of administration, like metronomic chemotherapy (mCHT), seem to be able to increase anticancer immunity, by acting on downregulation of Tregs. Most of the data available regarding the immunomodulating effect of mCHT have been obtained with Cyclophosphamide (CTX). Aim of the present study was to explore the effects of mVRL and mCAPE administration, alone or in combination, on T cells. Observation of 13 metastatic breast cancer patients lasted controlling for 56 days, where Treg frequencies and function, spontaneous anti-tumor T-cell responses were monitored, as well as the clinical outcome. No depletion in Treg absolute numbers, or percentage of T lymphocytes, was observed. Only in 5 patients, a modest and transient depletion of Tregs was observed during the first 14 days of treatment. To better describe the effect on Tregs, we subsequently looked at the variations in Memory, Naïve and Activated Treg subpopulations: we observed a trend in reduction for memory Treg (Treg MEM) and an increase for Treg Naïve (Treg NAIVE) and Treg Activated (Treg ACT) components. We finally analyzed the average trend of Treg in the Treg depleted patients and non-depleted ones, without fiding any significant differences. The trend of the Treg MEM appeared different, showing a reduction during the first 14 days, followed by an increase at the levels before treatment at Day 56 in the group of depleted patients and a progressive substantial reduction in the group of non-depleted patients along the entire course of treatment. Opposed to the data known, treatment with mVRL w/o mCAPE did not show any effect on Tregs.

**Supplementary Information:**

The online version contains supplementary material available at 10.1186/s12885-022-10031-6.

## Introduction

Breast cancer is the most common cancer in women. It is estimated that 685,000 women died worldwide in 2020 due to breast cancer, according to the World Health Organization (https://www.who.int/newsroom/fact-sheets/detail/breast-cancer).

Metastatic breast cancer (MBC) patients showed a median Overall Survival (OS) of 3–4 years, according to the different biologic intrinsic subtype.

Immunity plays an important role and severe immune suppression is frequent in late-stage tumor patients, promoting tumor immune evasion and subsequent tumor progression.

Cancer cells can be eliminated by the effector mechanisms of humoral and cellular immunity. Today, it is evident that both the innate immune system (Natural Killer, macrophages) and the adaptive one (CD8+ cytotoxic T lymphocytes and antibodies) can react against many cancers [1–4]. On the other hand, not only do many malignant neoplasms develop mechanisms that allow them to evade or resist antitumor responses, but it is now certain that the immune system itself, at times, promotes tumor growth. These mechanisms in general can be divided into intrinsic to neoplastic cells or extrinsic, that is, mediated by other cells. In particular, the interest will focus on the extrinsic mechanism, in which cells of the immune system mediate the suppression of antitumor immunity. Studies conducted on mouse models and cancer patients indicate that, in subjects with cancer, the number of regulatory T lymphocytes is increased and that these cells are localized in the leukocyte infiltrate characteristic of some types of cancer. Treg cells are CD4+/CD25+/FoxP3+ T lymphocytes, enriched with TNF (tumor necrosis factor) receptor and CTLA4 (cytotoxic T-lymphocyte-associated antigen-4) [5].

Treg cells are able of suppressing tumor-specific effector cells, such as CD8+ cytotoxic T lymphocytes, CD4+ T helper lymphocytes and NKcells. Tregs are in fact the cells responsible for the maintenance of immune peripheral tolerance. Their tolerogenic mechanism is known as suppression and consists in blocking the activation of the cytotoxic CD8+ T lymphocyte when it meets the dendritic cell [6].

High numbers of Tregs in peripheral blood and within the tumor tissues of patients with different types of cancer is associated with poor prognosis [7].

Different studies have demonstrated that some chemotherapeutic agents are able to induce short-lived, inhibitory effects on innate and adaptive immunity [4, 8, 9]. However, different drugs, and especially different schedules of administration, like metronomic chemotherapy, defined as the chronic administration of drugs at minimally toxic doses and with no prolonged drug-free breaks, seem to be able to increase anticancer immunity, especially acting on downregulation of Tregs [10–12].

Most of the data available regarding the immunomodulating effect of metronomic chemotherapy mCHT have been obtained with Cyclophosphamide (CTX).

In a study of MBC patients, 30 days of metronomic low-dose CTX treatment selectively depleted CD4+ CD25+ Foxp3+ Treg [10], suggesting that prolonged treatment with low-dose CTX may not only control immune suppression by Treg, but also support spontaneous anti-tumor T-cell responses and thereby clinical outcome of MBC patients.

Also, metronomic gemcitabine, paclitaxel and temozolomide have shown immunoregulatory properties acting on Tregs [13].

Other agents, such as VRL, a vinca alkaloid, or CAPE, a fluoropyrimidine, which are largely used in the treatment of MBC and widely studied in the metronomic schedule have been less explored [14–17]. No data are available now regarding a similar effect of mVRL w/o mCAPE.

In this exploratory Phase 2 study, we monitored for 3 months the effects of mVRL, with or without mCAPE, on Tregs frequencies and function, spontaneous anti-tumor T-cell responses, as well as the clinical outcome in a population of MBC patients undergoing mCHT.

## Materials and methods

### Patients and treatment

The study received the approval of the Comitato Etico Brianza. All patients provided informed consent before entering the study. All methods were performed in accordance with the relevant guidelines and regulations.

Main inclusion criteria were diagnosis of locally advanced or MBC, HER2 negativity according to ASCO/CAP guidelines, measurable or evaluable disease according to RECIST 1.1. criteria, hematologic, liver, and renal function within normal ranges, written informed consent.

Patients with known autoimmune diseases, presence of a second tumor different from breast cancer, previous or current intake of drugs with a known stimulatory effect on the immune system and previous treatment with VRL or CAPE were excluded.

Treatment consisted of mVRL at the dose of 50 mg three times per week, if administered alone, or at the dose of 40 mg thrice a week if administered together with CAPE at the dose of 500 mg thrice a day, continuously. All drugs were administered regularly from Day 1 until Day 56, which was considered the End of Study.

### Flow cytometry

The total analysis of lymphocytes and subpopulations was determined in accordance with what was observed by H. T. Maecker Maecker et al. (2000). The analysis of lymphocyte subpopulations was carried out using an 8-color BD FACS Canto™ II (Becton Dickinson, San Jose, CA) flow cytometer. The Treg lymphocyte subpopulation was identified by monoclonal antibodies labeled CD45 V500, CD3 V450, CD4 PerCP-Cy5.5, CD25 PE, CCR4 PE-Cy7, CD 27 Alexa 647, (BD Biosciences, San Jose, CA) and analyzed for medium of BD FACS Canto™ II (Becton Dickinson, San Jose, CA) using FACSDiva™ software.

Once the peripheral venous blood sample is drawn, it is then prepared for the flow cytometry analysis. To prepare the sample, 50 μl of blood and 50 μl of mixed antibodies are combined at room temperature, away from direct light sources. After about 30 minutes of incubation, time necessary for the binding between membrane antigens and antibodies, the erythrocyte component is eliminated using BD FACS™ Lysing Solution and subsequent two washes with PBS liquid and centrifugation at 1500 rpm for 5 minutes. The sample is then re suspended in PBS liquid for the flow cytometric analysis.

### Preparation of T cell

For each sample, 50,000 events were analyzed. Using the DIVA software, we delimited the populations under study from time to time with the gate function. To restrict the population of cells under study to the lymphocyte population excluding the other leukocytes, based on the physical parameters of FSC and SSC, the cell population with size and granularity characteristics compatible with the lymphocytes was selected (Supplementary Fig. 1a).

Subsequently, considering only the lymphocyte population, we selected the T component by enclosing in a gate only the population with high expression of CD3 V450-A. Then, within the CD3+ T lymphocyte population, we selected the CD4+ T cells by placing a gate that collects the cloud of cells with high CD4 expression (Supplementary Fig. 1b). We finally divided the CD4+ cells based on the expression of CCR4 PE-Cy7-A, thus selecting the CD4+ CCR4+, and within them the CD25 high/CD127low (Supplementary Fig. 1c).

Through this hierarchical cascade selection, based on physical characteristics and antibody expression, we were able to define the percentage of regulatory T cells present in the sample.

We also included in the antibody panel 45RO APC-H7 and CD 28 FITC (BD Biosciences, San Jose, CA), specific antibodies for molecules expressed by CD4 T lymphocytes, with the aim of discriminating between T memory, T naïve and activated T lymphocytes. We defined the positive (+) 45RO population as T memory cells, the negative (−) population as T Naïve cells. To correctly define the population of activated T lymphocytes, we chose CD28+ cells within the weakly positive CD45RO subpopulation.

In parallel to the measurements obtained with the 8 fluorescent antibodies, we recorded for each sample the analysis of the subpopulations in terms of percentage and absolute value (cells / mmc) of total lymphocytes, B lymphocytes, T3 lymphocytes, T4 lymphocytes, T8 lymphocytes and NK cells by standard 4-color flow cytometry, compliant with the National External Quality Assurance Scheme (NEQAS) standards.

By comparing the percentages obtained with the 8-color flow cytometer to the absolute value of T4 lymphocytes, we were able to trace the absolute value of the subpopulations of memory, naïve, activated T4 lymphocytes and of the total regulatory, memory, naïve and activated T lymphocytes.

### Statistical analysis

This is an exploratory Phase 2 study aiming at describing Treg frequencies and function under treatment with mVRL w/o mCAPE. Considering the exploratory design and the previous papers published on the same topic with similar numbers of patients, we decided to approach our hypothesis by using a similar sample size. If the effect of mVRL w/o mCAPE will be similar to that observed with metronomic CTX, we enroll up to 40 patients to further confirm our hypothesis.

The difference between the mean values of the Tregs at baseline and at days 14 and 56 were calculated using the two-tailed “Student’s t” test. This statistical test was chosen as the most appropriate due to the small sample size. A *p*-value < 0.05 was considered significant. The support of GraphPad Prism software version 7.01 was used for statistical analyses. All other analyses were conducted descriptively.

## Results

### Patient treatment and outcome

After the approval of Comitato Etico Brianza, between March and September 2016, we enrolled 13 consecutive MBC patients. Median age was 66.5 years (45–86); 2 patients had received mCHT as first-line treatment, the others as second- or further lines of treatment. Three patients were de-novo MBC.

Most of the patients were ER+ (11, 84.6%), had 2 or more metastatic sites (11, 86.4%), had received anthracyclines (7, 53.8%), taxanes (8, 61.5%), or both (2, 18.2%). Metastatic sites at the time of enrollment were mainly at bone (11, 84.6%), liver (5, 38.5%) and lung (3, 23.1%). Table 1 summarizes patients’ and disease characteristics.

**Table 1:** Patients’ and tumor characteristics at baseline

	Categoria	N.
**Age**	<65 years	6
	≥65 years	7
**Pathology**	Ductal carcinoma	11
	Lobular carcinoma	2
**ER**	Positive	11
	Negative	2
**PgR**	Positive	10
	Negative	3
**HER2**	Positive	0
	Negative	13
**Number of metastatic sites**	1	2
	2	5
	>2	6
**Line of treatment**	1	2
	≥2	11
**Previous anthracyclines**	Yes	7
	No	6
**Previous Taxanes**	Yes	8
	No	5
**Previous anthra and Taxanes**	Yes	2
	No	11
**Other previous treatments**	Yes	2
	No	11
**Type of mCHT**	mVRL	11
	mVRL + mCAPE	2

*Abbreviations*: *mCHT* metronomic chemotherapy, *mVRL* metronomic Vinorelbine, *mCAPE*, metronomic Capecitabine

Eleven patients (84.6%) were treated with mVRL at the dose of 50 mg three times per week, the remaining 2 with mVRL at the dose of 40 mg thrice a week plus CAPE 500 mg thrice a day. All drugs were administered in a continuous way from Day 1 until Day 56. No patient discontinued before the end of study and no dose reduction was required due to adverse events.

Blood cell count, together with liver and renal function were evaluated at baseline, after 2, 4 and 8 weeks (Day 56) of treatment. Two patients were discontinued before the end of evaluation at Day 56: The first patient due to deterioration of her clinical conditions, the second one was discontinued from the study due to the need to be treated with radiation therapy for pain at the lumbar spine. At the planned disease status restaging at Day 56, 1 patient obtained CR, 5 patients PR, 4 SD and only one patient showed disease progression (PD).

**Fig. 1:**
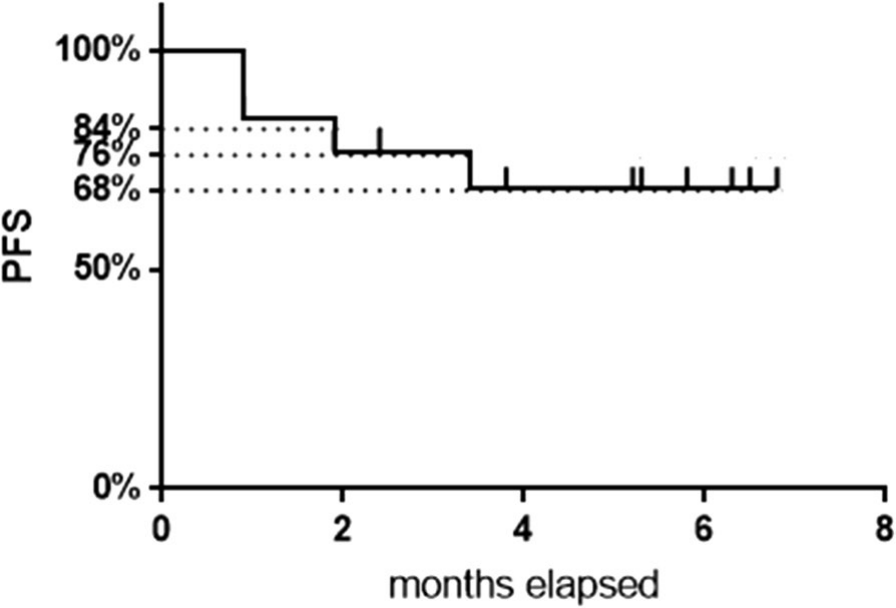
Progression Free Survival (PFS)

Patients who obtained a disease response continued the metronomic treatment: in these women, the disease response was confirmed at subsequent evaluations performed every two months of treatment. Only one patient died after four months of therapy due to a massive pulmonary thromboembolic event. Median PFS was 5.2 months (4–12) (Fig. 1).

### Effects of mCHT on Tregs

For each individual patient, we detected the level of circulating Treg both as a percentage of CD4 + lymphocytes and as an absolute value expressed in cells/mmc. In accordance with data available in literature the number of Tregs was increased in peripheral blood samples of cancer patients.

**Fig. 2:**
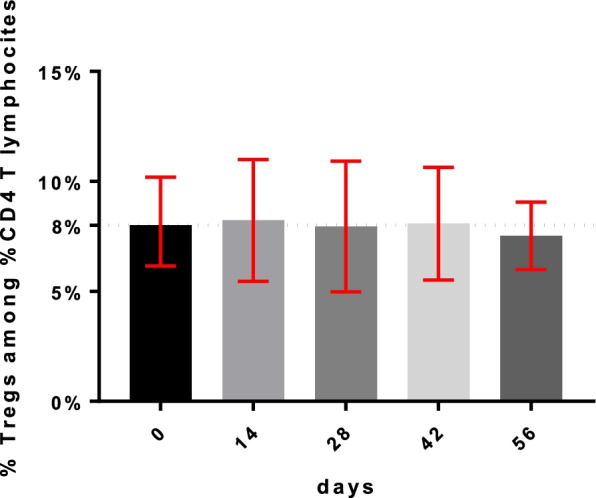
Treg trend during mCHT treatment as percentage

**Fig. 3:**
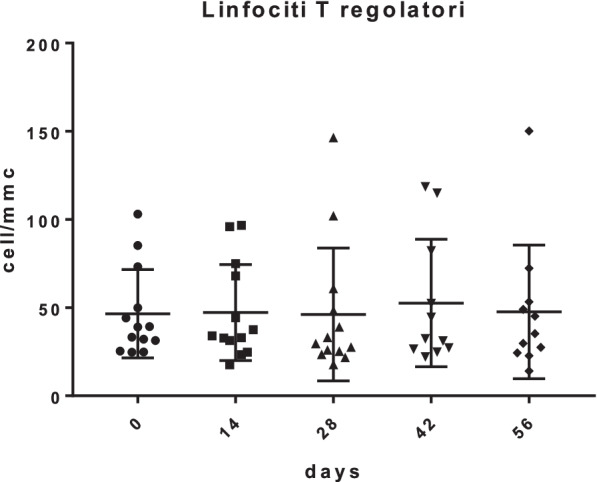
Treg trend during mCHT treatment as absolute value

The average level of Treg detected at T0 before the start of metronomic therapy was 8% of the CD4+ T lymphocytes, which corresponds to 46 cells/mmc as absolute value. Overall, mVRL did not seem to reduce Treg during treatment, as shown in Figs. 2, 3.

Mean values of Treg lymphocytes (total, memory, naïve and activated), expressed both as a percentage and as absolute value were stable without statistically significant increases or reductions. The same trend was found for all the other lymphocyte subpopulations analyzed such as total lymphocytes, B lymphocytes, T lymphocytes, CD8+ T lymphocytes and NK) cells. Only in 5 out of 13 patients (38.5%), a transient depletion from Day 1 to Day 14 was observed.

**Fig. 4:**
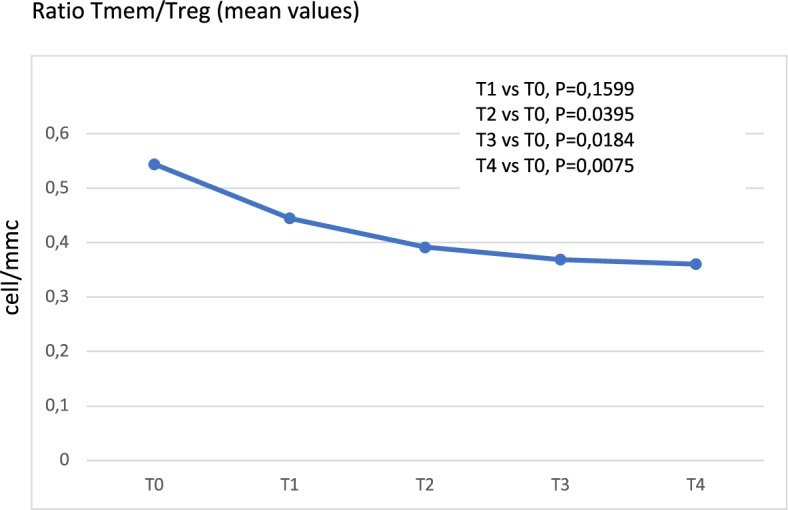
Ratios T subpopulations/Treg. Ratio Tmemory/Treg (mean values)

**Fig. 5:**
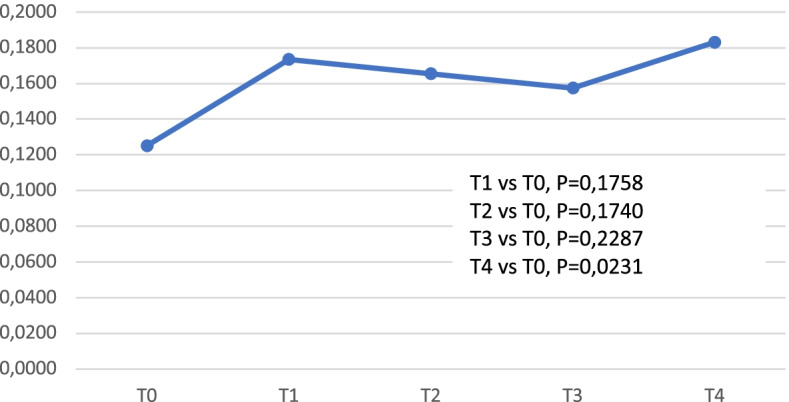
Ratios T subpopulations/Treg. Ratio T activated/Treg

Since the analysis of the trend of the average populations did not show significant fluctuations, we looked whether, within the population of Treg, there were variations in Memory, Naïve and Activated components. Even if the trend of these three subpopulations did not show any statistically significant results, comparing the average levels of the entire population over time, we observed a trend in reduction from T0 to T4 for Treg MEM and an increase for Treg NAIVE and Treg ACT. To discriminate the actual weight of each of these subpopulations within the total Treg lymphocytes, we analyzed the trend of the ratio Treg MEM / TREG, Treg Naïve / TREG and Treg Activated / TREG. As shown in Figs. 4 and 5, the trend of the Treg MEM / Treg proportion displayed a significant reduction from baseline until Day 56 (T4).

At the same time, the TregACT/Treg ratio showed a significant increase between T0 and T4 and the Treg Naive / Treg ratio shows a clear increasing trend, even if it did not reach statistical significance (T0/T3 *p*-value = 0.09). A trend in reduction was noted for Treg memory lymphocytes from 24.2 cells/mmc to 17.8 cells/mmc, which was not statistically significant; for naïve and activated Treg cells the trend was incremental, going from 5.6 cells/mmc to 10.4 cells/mmc and from 6.2 cells/mmc to 9.6 cells/mmc, respectively.

Finally, according to the presence or absence of Treg depletion after 14 days of treatment, patients were divided into two groups; for each group, the trend of the average levels of lymphocytes and of the T and NK subpopulations was further evaluated, without highlighting significant differences between the two groups (data not shown): however, in the group of patients with Treg depletion, a slight increase in the CD4 + population was observed, with substantial stability in the other cell populations, whereas, in patients without depletion, a minimal reduction in total lymphocytes was noted after 14 days of metronomic therapy.

**Fig. 6:**
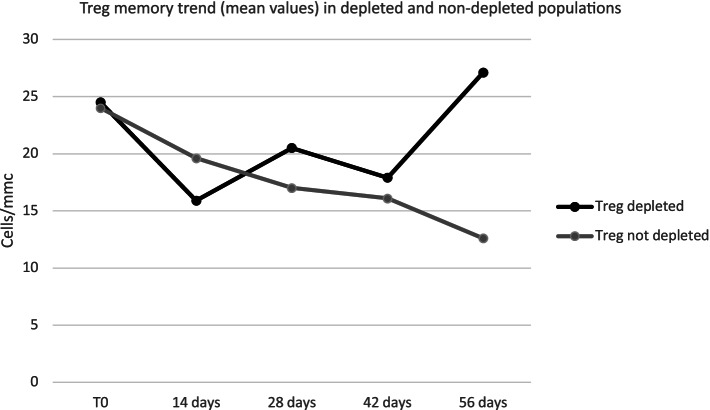
Treg MEM trend (mean values) in depleted and non-depleted populations

**Fig. 7:**
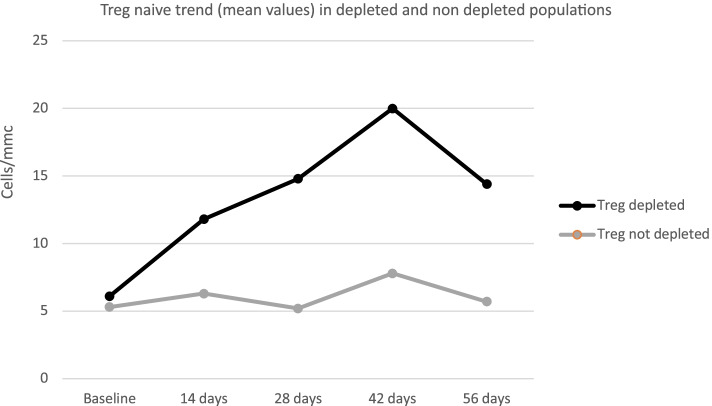
Treg naïve trend (mean values) in depleted and non-depleted populations

**Fig. 8:**
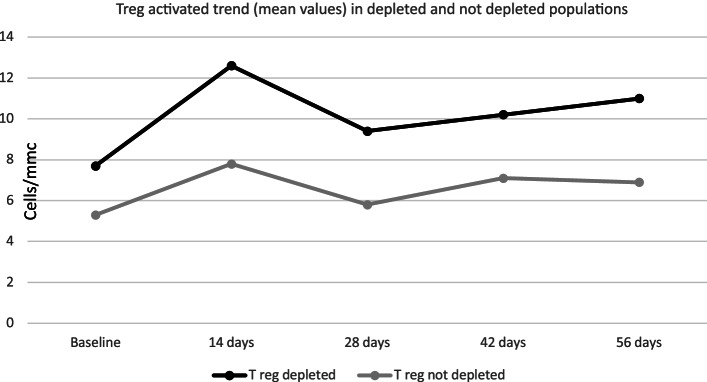
Treg activated trend (mean values) in depleted and not-depleted populations

For this reason, we further analyzed the average trend of Treg subpopulations (memory, naïve, activated) in these two different groups (Figs. 6, 7 and 8); no significant differences were found in the trend of Treg subpopulations: in both groups, the increasing trend of naïve Tregs and activated Tregs is comparable, however, the trend of the Treg memory appeared different, showing a reduction during the first 14 days, followed by an increase at the levels before treatment at Day 56 in the group of depleted patients and a progressive substantial reduction in the group of non-depleted patients along the entire course of treatment.

**Fig. 9:**
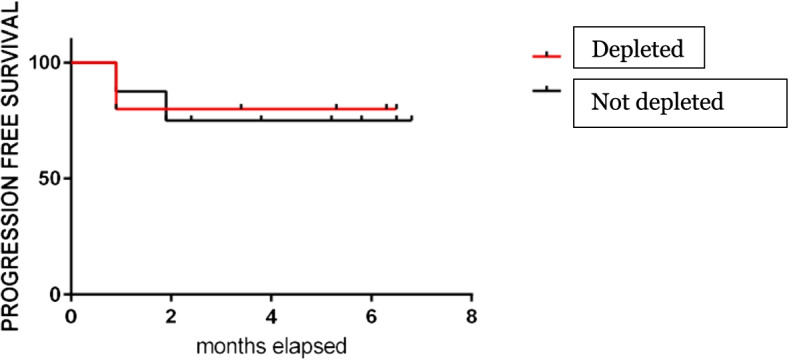
Progression Free Survival according to Treg depletion

We finally analyzed the PFS in the 2 populations according to depletion or not in Treg, without finding any significant difference (Fig. 9).

## Discussion

The immune system plays a crucial role in controlling the development of cancer.

The concept of immunosurveillance proposed in the 1950s by Burnet provides that cancer cells can be recognized and eliminated by the effector mechanisms of humoral and cellular immunity, innate (macrophages, Natural Killer) and adaptive (T and B lymphocytes).

As is known, most tumors still develop strategies that allow them to escape immune responses. It is now also established, that the immune system itself sometimes promotes tumor growth, through cells that mediate the suppression of antitumor immunity. Among these, a key role is played by regulatory T cells. Multiple studies conducted on mouse models and cancer patients have shown the presence of a high number of Treg lymphocytes in the peripheral blood of patients with cancer and in the leukocyte infiltrate of many neoplasms and that this correlates with the progression of the disease and with a worse prognosis. Recently, the identification of a unique lineage of CD4+ T cells, named Treg, with the capacity of suppressing T cell effector function has put a new light on the mechanisms of tumor escape [18]. Treg are also able to inhibit the proliferation of CD4+/CD25- and CD8+ lymphocytes: these two properties, taken together, represent a big risk for the equilibrium between formation and destruction of cancer cells: so far, it has become more and more important to investigate whether some drugs, or some schedules could exert an inhibitory effect on Treg population.

Preliminary preclinical and clinical studies suggested that CHT administered at the Maximum-Tolerated-Dose (MTD) on one side ablate cancer cells, but on the other side also could compromise the immune system [19], whereas mCHT can increase the ablation of immunosuppressive Treg cells [20–22].

Based on these preclinical data, Ge et Al. conducted a pilot study in 12 MBC patients, who received single agent CTX at the dose of 50 mg per day, with the aim of testing if a metronomic regimen of CTX was able to affect Treg population [10]. They showed that, after an initial substantial depletion of Treg in the first 14 days of treatment, Treg numbers completely recovered during the therapy. Despite the complete recovery of Treg at the end of observation, the increase in breast-tumor reactive T cells remained at high levels for the whole treatment. The Treg suppression exerted by CTX in almost all patients was maintained until Day 42, when Tregs gradually recovered until their complete restoration on Day 84. These data showed for the first time that a metronomic schedule of CTX was able to deplete Tregs, even if not in the longer period.

Orecchioni et Al. studied the effects of different doses of three orally active drugs (VRL, CTX and 5-FU) over the landscape of circulating and tumor-infiltrating immune cells involved in checkpoint inhibitors activity [23], as well as on local and metastatic tumor growth [24].

The Authors showed that VRL and CTX (at medium/high doses) reduced circulating Tregs. Cyclophosphamide (at low doses) and 5-FU (at medium doses) slightly increased circulating Tregs.

In the present study, we present data regarding the immunomodulatory effects of mVRL on Treg.

To our knowledge, this is the first study regarding the immune effects of mVRL, alone, or in combination with mCAPE.

In our series, mVRL did not seem to reduce Tregs during treatment: mean values of Tregs (total, memory, naïve and activated), expressed both as a percentage and as absolute value, were stable without statistically significant increases or reductions.

It is therefore potentially possible that VRL, albeit in a metronomic schedule, has a marginal immunomodulatory effect compared to other drugs such as cyclophosphamide, exerting its effects mainly on neo-angiogenesis and on the reduction in colony formation, as suggested by some pre-clinical data [23]. The increase in the pool of regulatory T lymphocytes after the first 14 days of therapy could be justified both by the increase in the naïve and activated Treg subpopulations and by the slight increase in the CD4+ T population in the group of depleted patients. In fact, tumor-related CD4+ T cells spontaneously produce IL-2, a cytokine essential for the development and activation of Tregs.

Orecchioni et Al. demonstrated that VRL at medium doses (6–9 mg/kg) and CTX at medium (20 mg/kg/day) and high (40 mg/kg/day) doses can reduce the number of circulating Treg in a tumor-bearing mouse model [23, 24]. It is possible therefore possible to argue that the doses of VRL used in our population (40 mg thrice a week in combination and 50 mg thrice a week as single agent) were too low to observe an immune effect on Tregs. In fact, the Optimal Biological Dose (OBD) of VRL was established at 9 mg/kg based on the capability of inhibiting CEC by 50%; so far, the OBD for a immunomodulatory effect could be different, with a clear consequence on the results observed in our population.

In fact, if the apoptotic action induced by VRL on endothelial cells with consequent inhibition of angiogenesis is rather known, there are no data that attest to the role played on immune suppression nor the possible dosage of drug capable of determining this effect. The timing of action in terms of immunosuppression of the two drugs may also be different and further studies are needed in this regard. It could also be useful to perform evaluations on the activity of the immune system in combination metronomic schemes such as VEX (Vinorelbine - Cyclophosphamide - Capecitabine) to observe and analyze the possible synergistic effect of chemotherapy drugs on different lymphocyte populations.

Finally, the analysis of total lymphocytes and of T lymphocytes and NK populations did not show a significant decrease over the time, confirming the literature data according to which metronomic chemotherapy does not exert a depletion of lymphocytes [25].

Interestingly, our data show that Treg memory trends are completely different in depleted vs non depleted groups of patients.

Recently, Treg cells have been subgrouped as either naive or effector/memory by their developmental stage and phenotype [26, 27]. Memory Treg cells are characterized by CD103 expression and the ability to home into inflammation sites. They have been proposed to be sentinels of tolerance, providing a first line of defense against potentially harmful inflammatory reactions.

Lin et al. [28] recently showed, using a concomitant immunity tumor model, that memory Treg cells increased as the progression of the primary tumor was observed [29]. Notably, these cells more greatly express killing molecules and suppress the functions of tumor-bearing CD8+ T cells in vitro and in vivo.

Moreover, other Authors [30] showed results like ours treating 23 MBC HR+ patients with exemestane + mCTX, they did not observe any treatment-related decrease in Tregs. Notably, in their study baseline Naïve Tregs were associated with 3-month PFS.

As expected, considering the small sample size, we didn’t observe any difference in terms PFS between depleted and not depleted patients.

## Conclusions

Tregs play an important role in tumor progression with several mechanisms. Therefore, it is evident that the control of these lymphocytes represents a key objective of oncological treatments.

Tregs show increased expression of FoxP3 that has been shown to repress the expression of several cytokines such as IL-2, IL-4 and IFNγ with concomitant activation of IL-2 receptor (CD25), cytotoxic T lymphocyte–associated protein-4 (CTLA4) and glucocorticoid-induced TNF receptor (GITR).

Metronomic chemotherapy, targeting Tregs, represents one of the possible therapeutic strategies.

In fact, many studies have shown that cyclophosphamide, administered at low-dose regimens, has a certain efficacy in terms of depletion of the population of regulatory T cells, while preserving other lymphocyte subsets, with consequent control of tumor progression.

Based on these findings we evaluated the immunomodulating action of vinorelbine with or without capecitabine administered with a metronomic schedule and on their activity on Tregs. Our study shows that the effect of such drugs on the immune system is only transient without statistically significance, suggesting that their action occurs mainly through other mechanisms. Further studies are needed in a larger population of patients, possibly divided into cohorts of different drug dosages, and with a longer observation period.

## Supplementary Information

**Additional file 1.**

## Data Availability

Not applicable.
