# Supplementary material for: Immunomodulatory effects of metronomic vinorelbine (mVRL), with or without metronomic capecitabine (mCAPE), in hormone receptor positive (HR+)/HER2-negative metastatic breast cancer (MBC) patients: final results of the exploratory phase 2 Victor-5 study
Source: BMC Cancer. 2022 Sep 6;22:956. doi: 10.1186/s12885-022-10031-6 (PMC9446532; doi:10.1186/s12885-022-10031-6)

**Supplementary Figures:**

**Figure 1 (a-b-c)** T cell identification.

1. Lymphocyte selection


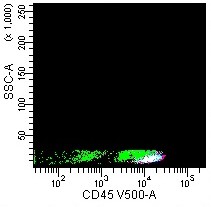

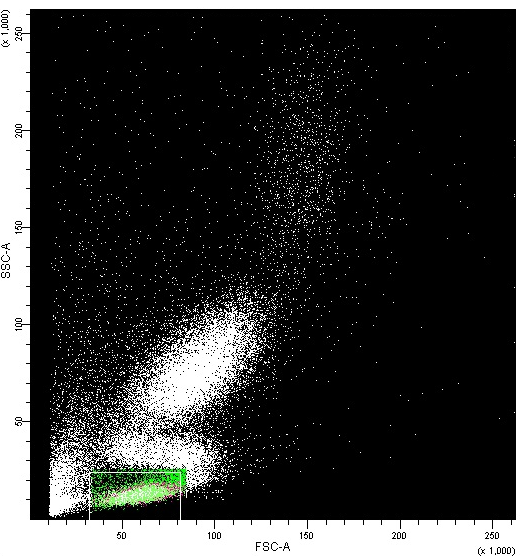


1. T lymphocyte identification and selection


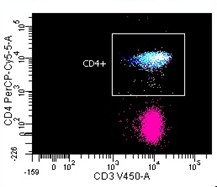

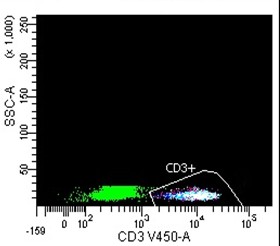


1. CD25+/ CD127- (Treg) identification


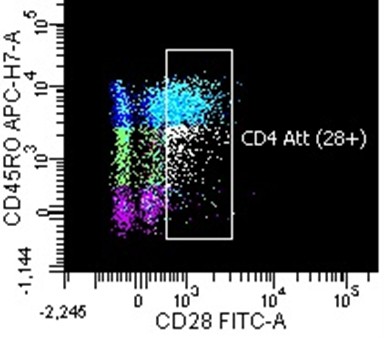

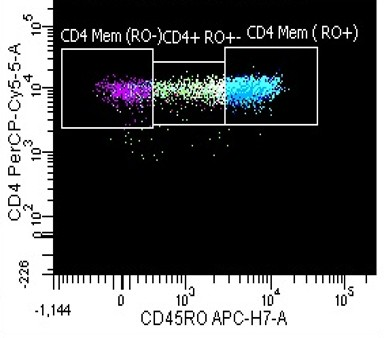

Supplement: Supplementary file 1 — Additional file 1. [file 12885_2022_10031_MOESM1_ESM.docx]
